# „Das muss man so nehmen.“ Eine Studie zum subjektiven Erleben der Coronapandemie älterer hilfe- und pflegebedürftiger Menschen in der Häuslichkeit

**DOI:** 10.1007/s00391-021-01888-6

**Published:** 2021-04-15

**Authors:** Angela Nikelski, Eva Trompetter, Stefanie Feldmann, Esther-Sarah Whittaker, Melanie Boekholt, Nino Chikhradze, Friederike Kracht, Petra Lücker, Horst Christian Vollmar, Jochen René Thyrian, Stefan H. Kreisel

**Affiliations:** 1 Abteilung für Gerontopsychiatrie und Forschungsabteilung der Klinik für Psychiatrie und Psychotherapie, Evangelisches Klinikum Bethel, Universitätsklinikum OWL der Universität Bielefeld, Campus Bielefeld-Bethel, Bethesdaweg 12, 33617 Bielefeld, Deutschland; 2 Deutsches Zentrum für Neurodegenerative Erkrankungen e. V. (DZNE), Standort Rostock/Greifswald, Greifswald, Deutschland; 3 Abteilung für Allgemeinmedizin, Ruhr-Universität Bochum, Bochum, Deutschland; 4 Institut für Community Medicine, Abt. Versorgungsepidemiologie und Community Health, Universitätsmedizin Greifswald, Greifswald, Deutschland

**Keywords:** COVID-19, Qualitative Forschung, Alltag, Bewältigung, Einsamkeit, COVID-19, Qualitative research, Everyday life, Coping, Loneliness

## Abstract

**Hintergrund:**

Da ältere Menschen ein erhöhtes Risiko für schwere und letale Verläufe einer SARS-CoV-2-Infektion aufweisen, erfahren sie besondere Aufmerksamkeit, die sich jedoch häufig einseitig auf ihre Schutzbedürftigkeit bezieht. Erforderlich ist eine Auseinandersetzung, die ihren subjektiven Wirklichkeiten Rechnung trägt und neben Risiken auch Ressourcen berücksichtigt.

**Ziel der Arbeit:**

Die Studie stellt die Perspektiven älterer Menschen in den Mittelpunkt, und Ziel ist es, ihr subjektives Erleben der Coronapandemie zu beleuchten. Gefragt wird danach, wie sie die Pandemie, Risiken, Folgen und Schutzmaßnahmen erleben, inwiefern sich diese auf ihren Lebensalltag auswirken und wie sie damit umgehen.

**Material und Methoden:**

Im Mai und Juni 2020 wurden 12 leitfadengestützte Telefoninterviews durchgeführt. Befragt wurden 9 Frauen und 3 Männer zwischen 77 und 91 Jahren, die in der eigenen Häuslichkeit leben, gesundheitlich beeinträchtigt, hilfe- und pflegebedürftig sind. Die Daten wurden mittels strukturierender Inhaltsanalyse ausgewertet.

**Ergebnisse:**

Die Befragten machen sich i. Allg. Sorgen aufgrund der Coronapandemie, schätzen die eigene Gefährdung aber als gering ein. Sie sehen sich von den unmittelbaren Folgen der Krise kaum betroffen oder stark in ihrem Alltag eingeschränkt. Unsicherheiten erleben sie im sozialen Lebensbereich, wobei die Angst vor Einsamkeit zentral ist. Sie halten die Schutzmaßnahmen insgesamt für angemessen, kritisieren aber frühzeitige Lockerungen, familiäre Kontaktbeschränkungen und den Umgang mit Sterbenden.

**Diskussion:**

Ein moderates Ausmaß unmittelbarer persönlicher Betroffenheit, Akzeptanz und Anpassungsfähigkeit prägen das Erleben und den Umgang älterer Menschen mit der Coronapandemie. Sichtbar werden Ressourcen und Kompetenzen im Umgang mit der Krise.

**Zusatzmaterial online:**

Zusätzliche Informationen sind in der Online-Version dieses Artikels (10.1007/s00391-021-01888-6) enthalten.

## Abstract (en)

**Background:**

As older people are at increased risk of severe and fatal courses of SARS-CoV‑2 infection, they receive special attention, which, however, often refers one-sidedly to their need for protection. What is needed is a discussion that considers the subjective reality and resources as well as risks.

**Objective:**

The study focused on the perspectives of older people. The aim was to shed light on their subjective experience of the corona pandemic. The questions were how they experienced the pandemic, the risks, consequences and protective measures, to what extent these affected their everyday life and how they dealt with it.

**Material and methods:**

A total of 12 guideline-based telephone interviews were conducted in May and June 2020 with 9 women and 3 men between 77 and 91 years of age, who lived in their own homes, had impaired health and needed help and care. The data were evaluated by structuring content analysis.

**Results:**

The respondents were generally concerned about the corona pandemic but considered their own risk to be low. They saw themselves as hardly affected by the immediate consequences of the crisis or severely restricted in their everyday lives. They experienced insecurity in social life and fear of loneliness was central. Overall, they considered the protective measures to be appropriate but criticized early cancelling, family contact restrictions and dealing with the dying.

**Conclusion:**

A moderate degree of direct personal involvement, acceptance and adaptability characterized the experience and handling of the corona pandemic. Resources and skills in dealing with the crisis become visible.

## Hintergrund und Fragestellung

Die Coronapandemie und die Maßnahmen zur Eindämmung des Infektionsgeschehens bestimmen seit Frühjahr 2020 das öffentliche und private Leben. Die Krise wirkt sich in unterschiedlicher Weise auf das Leben und den Alltag jedes Einzelnen aus. Anforderungen, Folgen und das Erleben der Pandemie variieren je nach Lebenssituation, was zielgruppenspezifische Analysen erforderlich macht. Nachfolgend stehen ältere Menschen im Mittelpunkt. Sie erfahren aktuell besondere Aufmerksamkeit, da sie aufgrund häufig bestehender Vorerkrankungen ein erhöhtes Risiko für schwere Verläufe einer SARS-CoV-2-Infektion und eine erhöhte Sterblichkeit aufweisen [6, 14]. Dies lässt nicht den Umkehrschluss zu, dass Personen ab einem bestimmten Lebensalter generell gefährdet sind, an COVID-19 zu erkranken. International werden kontroverse Diskussionen über altersspezifische Risiken und Schutzmaßnahmen geführt, die implizit oder explizit die gesellschaftliche Stellung älterer Menschen tangieren. Häufig wird ein Bild der „schwachen und schutzbedürftigen Alten“ entworfen, welches die Heterogenität dieser Gruppe kaum angemessen widerspiegelt. Im Gegenteil, derartig pauschalisierte Diskurse tragen u. U. dazu bei, Altersstereotype zu festigen und Altersdiskriminierung zu befördern [1, 11]. Wünschenswert ist ein Diskurs, der auch die Perspektiven älterer Menschen selbst und neben Risiken auch Ressourcen berücksichtigt. Befragungen älterer Personen tragen zu einem differenzierten Bild bei, wonach Ausmaß und Intensität der Sorgen und Ängste aufgrund von Corona und die Einschätzung der persönlichen Gefährdung innerhalb der Gruppe stark variieren [10, 12]. Kurzfristige Auswirkungen sozialer Kontaktbeschränkungen auf die psychosoziale Gesundheit konnten bislang nicht belegt werden [10, 12]. In einer Studie erwies sich das höhere Lebensalter als protektiver Faktor, was die Vermutung nahelegt, dass Ältere besser in der Lage sind, mit kritischen Lebenssituationen umzugehen [9]. Wollen wir den Umgang älterer Menschen mit der COVID-19-Pandemie besser verstehen, ist es erforderlich, ihre Erfahrungen in den Mittelpunkt zu rücken. Dieses Anliegen verfolgt die vorliegende Studie, in der Menschen im höheren Lebensalter danach gefragt werden, wie sie die Pandemie, die damit einhergehenden Risiken, Folgen und Schutzmaßnahmen erleben, inwiefern sich diese auf ihren Lebensalltag auswirken und wie sie damit umgehen.

## Studiendesign und Methoden

Gewählt wurde ein exploratives qualitatives Studiendesign. Im Mai und Juni 2020 wurden 12 leitfadengestützte Telefoninterviews mit Personen zwischen 77 und 91 Jahren (Ø 82,3 Jahre), die in der eigenen Häuslichkeit leben, durchgeführt (Interviewdauer: 18–50 min). Die Stichprobe setzt sich mehrheitlich aus Frauen zusammen (*n* = 9), und etwas mehr als die Hälfte lebt alleine (*n* = 7) (Zusatzmaterial online: Stichprobenbeschreibung). Die Rekrutierung erfolgte im Rahmen der Interventionsstudie „intersec-CM: Intersektorales Care Management – Unterstützung älterer Menschen mit kognitiver Beeinträchtigung während und nach dem Krankenhausaufenthalt“ [7]. Eine kognitive Beeinträchtigung während eines Krankenhausaufenthaltes war ein Einschlusskriterium in die oben genannte Hauptstudie, ist aber kein Kriterium, welches die Gruppe dieser qualitativen Studie spezifiziert, zumal die nun Befragten nur leichte Einschränkungen aufweisen. Maßgeblich charakterisiert wird die Zielgruppe durch einen eingeschränkten Gesundheitszustand sowie die vorliegende Hilfe‑/Pflegebedürftigkeit. Die Teilnahme an der zusätzlichen Befragung zu Corona war freiwillig und basierte auf einer informierten schriftlichen Einwilligung. Die Datenerhebung erfolgte mittels problemzentrierter Interviews [13]. Eingesetzt wurde ein Gesprächsleitfaden, der die subjektive Sicht der Befragten in den Mittelpunkt stellte. Anhand eines Kurzfragebogens wurden soziodemografische Daten erhoben. Für jedes Gespräch wurde ein Postskript erstellt. Die Interviews wurden digital aufgezeichnet, transkribiert [2] und mittels inhaltlich strukturierender Inhaltsanalyse mit deduktiv-induktiver Kategorienbildung ausgewertet [5] (Zusatzmaterial online: Datenerhebung und Datenauswertung). Die Ergebnisse werden nachfolgend anhand 7 zentraler Themenbereiche vorgestellt.

## Ergebnisse

### Corona: Ich habe schon Schlimmeres erlebt

Die befragten Personen sind erschrocken über die Coronapandemie und berichten, nie etwas Ähnliches erlebt zu haben. Gleichzeitig versuchen sie, die Pandemie in einen Kontext einzuordnen. Hergestellt werden vorsichtige Vergleiche mit Erkrankungen, wie der Spanischen Grippe, Poliomyelitis, der Grippe oder auch Ereignissen wie dem Zweiten Weltkrieg. Insbesondere persönliche Erinnerungen an die Kriegszeit, die durch Bombenangriffe, Flucht, Hunger, „Lebensmittelhamstern“ und den Wiederaufbau gekennzeichnet waren, sind wieder präsent. Viele Teilnehmer*innen erzählen, zeitlebens hart gearbeitet und Entbehrungen ertragen zu haben. Das Wissen, schon „Schlimmeres“ erlebt (TN01; TN = Teilnehmer*in) und „schlimmere Zeiten“ (TN12) überstanden zu haben, stärkt sie im Umgang mit der Pandemie und den dadurch bedingten Einschränkungen. Auch wenn sie sich Gedanken etwa über die Entstehung und Beherrschbarkeit des Virus, die Leistungsfähigkeit des Gesundheitssystems, die Impfstoffentwicklung und eine mögliche Impfpflicht sowie über die Pandemiedauer machen, beschreiben sie gleichzeitig eine Situation, die auszuhalten ist und an die man sich anpassen kann und muss. Der Rückblick auf zurückliegende Lebensereignisse, die eigene Machtlosigkeit sowie die empfundene generationsbedingte Genügsamkeit tragen dazu bei, die momentane Situation für sich selbst gut akzeptieren zu können.

### Gefährdung: Wenn es mich trifft, trifft es mich

Die Gesprächspartner*innen sind besorgt, dass Angehörige oder Bekannte erkranken könnten und geben an, dass sie selbst aufgrund ihres Alters und bestehender Vorerkrankungen zu einer Risikogruppe gehören. Dieses Wissen wird sowohl medial als auch durch Personen im privaten Umfeld vermittelt. Insbesondere jüngere Familienmitglieder mahnen zur Vorsicht. Nach anfänglicher Unsicherheit hinsichtlich der Ansteckungsgefahr sowie möglicher Folgen sieht sich aktuell niemand der Befragten größeren Gefahren ausgesetzt. Die subjektiv empfundene Gefährdung, sprich die eigene Angst, sich zu infizieren, zu erkranken und zu versterben, ist gering, wird geschmälert oder hingenommen. Auch hier kommt erfahrungsbedingt die Gewissheit zum Tragen, mit schwierigen Situationen umgehen zu können. Zentrale Erklärung für die ausbleibende Sorge um sich selbst ist die aktuelle Lebenssituation, die durch einen beeinträchtigten Gesundheitszustand (Immobilität, Schmerzen etc.) geprägt ist. Da soziale Kontakte und Aktivitäten außer Haus dadurch häufig eingeschränkt sind, wird das Ansteckungsrisiko als gering erachtet. Zudem erfordert die tägliche Bewältigung gesundheitlicher Probleme so viel Anstrengung, dass sie sich mit wenig zufriedengeben und sich grundsätzlich als dankbar beschreiben. Das erreichte Lebensalter und das nahende Lebensende lassen sowohl den Zeitpunkt des Todes als auch die Ursache – und damit eben auch COVID-19 – weniger relevant erscheinen. Dem Tod – sei er Schicksal oder Zufall – sehen sie sich machtlos gegenüber. Ziel ist es, wie gewohnt weiterzuleben und die verbleibende Lebenszeit zu genießen. Sie sehen sich in keiner besonderen Position als älterer Mensch, erkranken und versterben doch Personen aller Altersgruppen.

### Folgen der Coronakrise: Ich bin nur Zuschauerin

Mit Ausnahme einer Teilnehmerin, die von Existenzängsten aufgrund der ausbleibenden finanziellen Unterstützung durch den Sohn berichtet, sieht sich kaum jemand unmittelbar mit den Auswirkungen der Coronakrise konfrontiert. Finanz- und Versorgungssicherheit bleiben von dieser weitestgehend unberührt. Stärker betroffen sind ihrer Meinung nach Erwerbstätige, Familien, Kinder, Alleinerziehende, Menschen in Armut, Pflegeheimbewohner*innen und Obdachlose, die vor größeren Herausforderungen als sie selbst stehen und drastische Einschränkungen hinnehmen müssen. Finanzielle Hilfen und Unterstützungsangebote für diese Gruppen werden befürwortet. Befürchtet wird eine wirtschaftliche Krise, die ihren Ausdruck bereits in der gefühlten Verteuerung von Lebensmitteln sowie in Geschäftsschließungen findet und die die Gesellschaft weiterhin spalten und existierende Ungerechtigkeiten befördern könne. Persönliche Herausforderungen stehen nicht im Kontext der Pandemie, sondern beziehen sich auf die Bewältigung von Krankheiten sowie die Auseinandersetzung mit der zunehmenden Hilfebedürftigkeit und der Sicherung alltäglicher Unterstützung.

### Alltagsleben: Mein Leben ist jetzt nicht so anders

Der Lebensalltag hat sich bei den wenigsten Befragten merklich verändert. Begründet wird dies mit einer an das Lebensalter und den Gesundheitszustand angepassten Lebensweise. Zugrunde liegende Erkrankungen und Mobilitätseinschränkungen gehen mit einer Konzentration auf das eigene Zuhause einher. Aktivitäten außer Haus und soziale Kontakte sind altersbedingt weniger geworden. Für die meisten Gesprächspartner*innen ist es normal, viel Zeit im eigenen Zuhause zu verbringen, und nur wenige fühlen sich im Zuge der Pandemie verstärkt an die Häuslichkeit gebunden. Gleichzeitig erfüllt das eigene Zuhause die Funktion eines Schutzraumes, in den sie sich freiwillig zurückziehen. Aktivitäten außer Haus werden auf das Nötigste und für sie Wichtigste reduziert, wozu Einkäufe, Friedhofs- und Arztbesuche zählen. Routinierte Abläufe und der normale „Alltagstrott“ (TN09) werden größtenteils beibehalten. Die Mehrheit der Befragten wird von Angehörigen, privaten und professionellen Diensten bei alltäglichen Verrichtungen unterstützt. Derartige Unterstützungsleistungen werden nahezu uneingeschränkt fortgeführt. Lediglich eine Teilnehmerin hat zeitweise freiwillig auf Hilfe im Haushalt verzichtet. Die Personen, die alleine leben und sich selbstständig versorgen, wurden zu Pandemiebeginn durch Lebensmitteleinkäufe von ihren Kindern unterstützt. Mittlerweile übernehmen sie diese wieder selbst, minimieren aber die damit verbundenen Gefahren, indem sie seltener einkaufen gehen und weniger Geschäfte als sonst üblich aufsuchen. Eingeschränkt fühlen sie sich dadurch nicht. Im Bereich der gesundheitlichen Versorgung berichten sie kaum von Einschnitten. Rehabilitationssport und Physiotherapie sind kurzzeitig ausgefallen, ärztliche und pflegerische Dienste werden aber wie gewohnt angeboten und wie üblich in Anspruch genommen. Optimierte Abläufe (kürzere Wartezeiten, postalischer Rezeptversand) verbessern die Versorgung stellenweise.

### Familiäre Kontakte: Das ist es doch, wofür ich lebe

Einschränkungen und Unsicherheiten erleben die Interviewteilnehmer*innen im sozialen Bereich. Zu Beginn der Pandemie wurden persönliche Treffen innerhalb der Familie kurzzeitig, in erster Linie aus Sorge der Jüngeren um die Älteren, eingestellt oder aber durchgehend beibehalten. Familiären Kontaktbeschränkungen begegnen die Befragten vernunftbasiert mit Einsicht und Verständnis, erleben diese gleichzeitig aber als starke Belastung und erhebliche Einschränkung der Lebensqualität. Das Zusammensein mit Angehörigen, v. a. mit den Kindern, ist für sie Teil einer sinnhaften Lebensgestaltung. Fallen diese Begegnungen weg, sind nur bedingt möglich oder potenziell in Gefahr, wird dies als Verlust oder Bedrohung wahrgenommen. Zentrale Themen sind die Angst vor Einsamkeit und die Sorge, zukünftig alleine zu sein sowie ggf. alleine zu sterben. Ein Wegfall familiärer Kontakte kann nicht adäquat kompensiert werden. Dass Treffen mit Bekannten und organisierte Freizeitaktivitäten ausbleiben, wird bedauert, aber hingenommen. Zur Kontaktpflege nutzen die Befragten das Telefon, und nur wenige gebrauchen darüber hinaus digitale Anwendungen, wobei Kontakte zu entfernteren Bekannten auf diese Weise kaum aufrechterhalten werden können. Der vielfach geäußerte Wunsch zur ursprünglichen Normalität zurückzukehren, bezieht sich v. a. auf den Bereich des sozialen Lebens.

### Information: Ich weiß, was ich wissen muss

Die Befragten nutzen Zeitung, Fernsehen und Radio als Informationsquellen, und der Großteil von ihnen fühlt sich mit Blick auf erforderliches Basis- und Handlungswissen ausreichend und verständlich informiert. Teilweise wird ein Informationsüberfluss bemängelt, hat das eigene Interesse an der Thematik doch im Laufe der Zeit nachgelassen. Unsicherheiten und Skepsis beziehen sich auf die Vollständigkeit und die Zeitpunkte der erhaltenen Informationen. Die Meinungsvielfalt, darunter auch Verschwörungstheorien, irritieren und verunsichern sie. Dies begründet einerseits ihren Wunsch nach mehr Klarheit und Verständlichkeit und erfordert andererseits eine selbstständige Meinungsbildung, wozu sie sich uneingeschränkt in der Lage sehen.

### Infektionsschutz: Daran kann man sich größtenteils halten

Grundsätzlich befürworten die Befragten die Infektionsschutzmaßnahmen und halten diese für richtig und angemessen. Auch wenn sie manche Diskussionen darüber für schwer nachvollziehbar halten und sich mehr Einheitlichkeit und Sorgfalt politischer Entscheidungen wünschen, schätzen sie das Vorgehen hierzulande als besonnen und erfolgreich ein. Man müsse auf sich selbst aufpassen, und in den meisten Situationen halten sie die empfohlenen Maßnahmen ein. Diese sind zur Routine geworden, und es fällt ihnen leicht, die Vorschriften zu verstehen, zu akzeptieren und umzusetzen. Beeinträchtigungen werden durch das Tragen eines Mundschutzes erlebt, wodurch Sehen, Hören und Verstehen zusätzlich erschwert sind. Besorgt nehmen sie wahr, dass sich aus Unvernunft, Uneinsichtigkeit und Erschöpfung nicht alle an die Regeln halten und wünschen sich gesellschaftliche Solidarität und Durchhaltevermögen. Bedenken werden hinsichtlich zu früher Lockerungen geäußert. Um sich selbst und andere zu schützen, schränken sie sich weiterhin freiwillig ein, verzichten z. B. auf Treffen mit Bekannten und bleiben überwiegend zu Hause. Für unverhältnismäßig halten sie demgegenüber strikte Maßnahmen innerhalb der eigenen Familie, und so haben viele die Entscheidung getroffen, auf die Einhaltung dieser zu verzichten. Persönliche Zuwendung und körperliche Nähe sind wichtiger als der Infektionsschutz. Vor diesem Hintergrund werden auch Besuchsverbote in Krankenhäusern und Pflegeeinrichtungen und der damit einhergehende „unmenschliche“ (TN10) Umgang mit alten, kranken und sterbenden Menschen kritisiert – Kontaktlosigkeit und ohne Begleitung zu sterben, seien schließlich schlimmer als Corona. Abgesehen von diesen als überzogen bewerteten Vorkehrungen nehmen sie ein politisches Bemühen um Ältere im Sinne einer Wertschätzung zur Kenntnis, die sie ansonsten oft vermissen, z. B. bei der Bekämpfung von Altersarmut. Gleichzeitig signalisieren sie, dass Ältere keinen besonderen Schutz oder spezielle Hilfen benötigen, müssen sie ihrer Ansicht nach doch vergleichsweise mit weniger Einschränkungen zurechtkommen als andere und sehen sie sich besser dazu in der Lage, sich anzupassen.

## Diskussion

Die Studie gewährt Einblicke, wie die befragten Älteren die Coronapandemie und die Auswirkungen auf ihren Lebensalltag erleben und damit umgehen. Ausgehend von den subjektiven Wirklichkeiten wird sichtbar, dass die Befragten i. Allg. zwar besorgt sind, Erleben und Umgang insgesamt aber durch ein moderates Ausmaß unmittelbarer persönlicher Betroffenheit sowie durch ein hohes Maß an Akzeptanz und Anpassungsfähigkeit geprägt sind. Erleben und Bewältigung sind vor dem Hintergrund der Lebenssituation und Biografie zu betrachten. Kritische Lebensereignisse und Krisen überstanden zu haben bzw. sich täglich mit gesundheitlichen Beschwerden und Selbstständigkeitsverlusten auseinanderzusetzen, sind Erfahrungen, die nun als Vergleichsparameter herangezogen und als Ressourcen wirksam werden. Eine an die gesundheitlich bedingten Einschränkungen angepasste Lebensweise sowie das nahende Lebensende lassen das Erkrankungsrisiko gering und kaum bedeutsam erscheinen. Damit steht das vermittelte und objektiv bekannte Risiko einer subjektiv als gering empfundenen Gefährdung gegenüber. Inwieweit diese Gelassenheit durch eigene oder gesellschaftliche Altersbilder beeinflusst wurde, kann lediglich vermutet werden. Gehört es zum eigenen Altersbild, sich als genügsam, zurückhaltend und in Anbetracht der Lebenserfahrung als resistent und anpassungsfähig zu beschreiben? Aufschlussreich wären weiterführende Studien, die sich mit altersbedingten Rollenbildern im Kontext der Pandemie beschäftigen. Sorgen i. Allg. und konkret um andere, z. B. um Angehörige, scheinen oft größer als die Sorge um sich selbst, was sich auch in anderen Studien andeutet [3, 10, 12]. Vielleicht bleibt das Bedrohungserleben auch deshalb abstrakt, weil Infektionen im nahen Umfeld kaum bekannt sind [10, 12]. Unabhängig von der eigenen Gefährdung wird die Pandemie aber grundsätzlich als bedrohlich erlebt und aus einer übergreifenden Perspektive betrachtet. Ängste und Unsicherheiten beziehen sich auf das direkte Pandemiegeschehen und gesellschaftliche Folgen. Im Vergleich zu Jüngeren und in prekären Verhältnissen lebenden Gruppen fühlen sich die Älteren selbst von den Folgen der Krise weniger betroffen und schätzen ihren eigenen Unterstützungsbedarf durch die Politik als gering ein. Der Informationsstand wird als gut und die Maßnahmen zur Eindämmung des Infektionsgeschehens werden grundsätzlich als angemessen beurteilt. Die Befragten akzeptieren die Schutzmaßnahmen größtenteils und sind skeptisch gegenüber geplanten Lockerungen, die ihrer Ansicht nach eine Verschlechterung der Lage begünstigen. Sie begegnen dieser Sorge mit Zurückhaltung und freiwilligen Einschränkungen. Ein guter Informationsstand sowie eine mehrheitliche Zustimmung zu den Schutzvorkehrungen ist bekannt [10, 12]. Anschlussfähig sind auch die Befunde zu den geringen Veränderungen des Alltagslebens [12]. Auswirkungen auf tägliche Routinen sind überschaubar, und gleichzeitig wird an Gewohnheiten festgehalten, die Stabilität vermitteln. Die Befragten fühlen sich, mit Ausnahme des sozialen Lebens, in ihrer Lebensführung kaum eingeschränkt, was auf den Gesundheitszustand und den daran angepassten Lebensstil zurückzuführen ist. Konkret begründet wird dies mit bekannten Phänomenen, wie der zunehmenden Konzentration auf das Zuhause und dem Rückgang sozialer Netzwerke [4, 8]. Das eigene Zuhause ist sicherer und freiwilliger Rückzugsort und fungiert auch in Coronazeiten als Schutzraum, wobei sich die eigene Wohnung über alle Altersgruppen hinweg als primärer Sicherheitsraum erweist [3]. Zugang zum gesundheitlichen Versorgungssystem und Inanspruchnahmeverhalten sind stellenweise tangiert, die Einschränkungen fallen aber insgesamt moderat aus, was vorangegangenen Befragungen entspricht [10, 12]. Die Familie spielt eine zentrale Rolle bei der Sicherstellung der alltäglichen Versorgung, indem die Angehörigen neben emotionaler Unterstützung eine Vielzahl praktischer Hilfeleistungen erbringen. Auch Röhr et al. [10] verweisen auf das hohe familiäre Unterstützungspotenzial. Weitere Studien zur Perspektive pflegender Angehöriger, die Aufschluss über Kompensationsleistungen, Belastungen und Folgen geben, sind wünschenswert. Wahrgenommene Einschränkungen und Ängste konzentrieren sich auf den sozialen Lebensbereich, und sichtbar wird hier die ambivalente Haltung gegenüber empfohlenen Schutzvorkehrungen. Prioritäten der Befragten bestehen darin, tägliche Herausforderungen zu meistern und die verbleibende Lebenszeit sinnvoll und lebenswert zu gestalten. In diesem Kontext spielen familiäre Beziehungen eine zentrale Rolle, die sie durch die Pandemie und präventive Maßnahmen in Gefahr sehen. Nicht zuletzt deshalb treffen viele die Entscheidung, der persönlichen Nähe den Vorrang zu geben. Die würdevolle Gestaltung des Lebensendes ist ein zentrales Thema, das die Befragten unmittelbar berührt, und die Vorstellung, alleine sterben zu müssen, erleben sie als greifbare Bedrohung.

Die Erkenntnisse schließen an vorliegende Befunde an und tragen aufgrund des qualitativen Ansatzes zu einem umfassenderen Verständnis bei. Die Auseinandersetzung mit den Lebenswirklichkeiten lenkt den Blick auf interne und externe Ressourcen sowie auf Kompetenzen im Umgang mit der Krise. Die Befragten sehen sich aufgrund ihres Lebensalters weder in einer herausragenden gesellschaftlichen Stellung noch als besonders betroffen, schwach oder schutzbedürftig an. Sie stellen sich selbst nicht in den Mittelpunkt, setzen sich auf einer gesellschaftlichen Ebene kritisch mit dem Pandemiegeschehen auseinander und heben ihre Fähigkeiten hervor, sich selbst schützen und sich eigenverantwortlich, selbstbestimmt und geduldig an die Gegebenheiten anpassen zu können. Akzeptanz und Anpassungsfähigkeit sind sowohl der empfundenen Machtlosigkeit geschuldet als auch biografisch begründet, gab es doch auch schwere Krisen in der Vergangenheit und fordert sie aktuell die Bewältigung von Krankheiten und Autonomieverlusten. Der stellenweise fast schon souverän anmutende Umgang wird dann erschüttert, wenn sie die wenigen Prioritäten und verbleibenden Lebensziele, wie die Nähe zur Familie und ein würdiges Lebensende, in Gefahr sehen. Einsamkeit ist demnach eine größere Bedrohung als Corona.

Ältere Menschen sind keine homogene Gruppe, und so sind auch die Ergebnisse nur bedingt auf ältere Menschen i. Allg. übertragbar. Betrachtet wurde eine spezifische Gruppe, die durch gesundheitliche Beeinträchtigungen, Hilfe- und Pflegebedürftigkeit gekennzeichnet ist und damit im Rahmen der Coronapandemie als besonders gefährdet gilt. Weder während der Durchführung der Interviews noch bei der Analyse des Datenmaterials gab es Hinweise darauf, dass eine zurückliegende oder ggf. noch bestehende kognitive Beeinträchtigung eine entscheidende Rolle spielte. Während innerhalb der Stichprobe eine Kontrastierung hinsichtlich subjektiver Gesundheit, Grad der Selbstständigkeit, Alter, Geschlecht und Lebenssituation erreicht wurde, sind die Ergebnisse z. B. durch die Unterrepräsentanz von Männern und die Sample-Größe limitiert. Mit Blick auf die individuelle Teilnahmebereitschaft sind Selektionseffekte nicht ausgeschlossen, und es ist davon auszugehen, dass vorwiegend diejenigen erreicht wurden, die aufgrund der COVID-19-Pandemie nicht in eine schwere persönliche Krise geraten sind.

## Fazit für Forschung und Praxis

- Erleben und Bewältigung von Krisen sind lebensphasenspezifisch und biografisch geprägt. Für die Forschung ergibt sich daraus die Notwendigkeit zielgruppenspezifischer Analysen. Individuelles Fallverstehen sollte für jede Profession, die mit älteren Menschen arbeitet, handlungsleitend sein.
- Ältere Menschen verfügen über Ressourcen und Kompetenzen, sich mit der Pandemie auseinanderzusetzen und sich in geduldiger Weise an die Gegebenheiten anzupassen. Die einseitige Betonung von Vulnerabilität und Schutzbedürftigkeit ist nicht angebracht und birgt die Gefahr, Selbstbestimmung und Handlungsfähigkeit unangemessen zu schmälern.
- Die Angst vor Einsamkeit hat sich als eine zentrale Sorge herauskristallisiert. Einsamkeit zu verhindern und soziale Teilhabe zu gewährleisten, sind zentrale Aufgaben. Die Begleitung sterbender Menschen ist unter ethischen Gesichtspunkten zu diskutieren.
- Die kontinuierliche (Selbst‑)Reflexion existierender Rollen- und Altersbilder kennzeichnet professionelles Arbeiten in Forschung und Praxis.
- Politische Entscheidungen sind kritisch dahingehend zu prüfen, inwieweit sie Ausgrenzung und Stigmatisierung aufgrund des Lebensalters begünstigen. Alle Mitglieder einer Solidargemeinschaft tragen die Verantwortung, sich selbst und andere zu schützen. Lebensalter und Generationenzugehörigkeit sind keine Kriterien, wonach sich diese Aufgabe bemisst.

## Supplementary Information




