# Supplementary material for: “You have to take it that way.” A study of the subjective experience of the corona pandemic by older people in need of help and care living at home
Source: Z Gerontol Geriatr. 2021 Apr 15;54(4):359–64. [Article in German] doi: 10.1007/s00391-021-01888-6 (PMC8047544; doi:10.1007/s00391-021-01888-6)
Supplement: Supplementary file 1 [file 391_2021_1888_MOESM1_ESM.docx]

**„Das muss man so nehmen.“ Eine Studie zum subjektiven Erleben der Coronapandemie älterer hilfe- und pflegebedürftiger Menschen in der Häuslichkeit**

**"You have to take it that way." A study of the subjective experience of corona pandemic of older people in need of help and care living at home**

**Supplement: Datenerhebung und Datenauswertung**

**Methoden: Datenerhebung und Datenauswertung**

Die Datenerhebung erfolgte mittels problemzentrierter Interviews [15]. Vor diesem Hintergrund wurden folgende Instrumente eingesetzt:

***Interviewleitfaden:*** Mit dem Ziel, Informationen strukturiert erfassen und auswerten zu können, wurden leitfadengestützte Interviews geführt. Die Fragestellung wurde im Vorfeld anhand eines Interviewleitfadens operationalisiert und umfasste die Themenbereiche „Coronapandemie“ (Risikoerleben, Schutzmaßnahmen, Schutzverhalten, politisches Handeln, Information) und „Alltagsleben“ (Auswirkungen auf verschiedene Lebensbereiche, Sozialleben, Gesundheit, Gesundheitsversorgung, Alltagsversorgung, Hilfe- und Unterstützungsleistungen aus dem privaten und professionellen Umfeld, alltägliche Sorgen und Herausforderungen). Der Leitfaden setzte sich aus Erzählanreizen, konkreten Fragen sowie Detail- und Vertiefungsfragen zusammen und wurde, ebenso wie Kommunikationstechniken, flexibel eingesetzt.

***Kurzfragebogen:*** Anhand eines Kurzfragebogens wurden folgende Daten der Studienteilnehmer*innen erfasst: Alter, Geschlecht, Familienstand, Schul- und Berufsbildung, Geburtsort, Wohnort, Wohnsituation. Um den Gesundheitszustand sowie den Hilfe- und Unterstützungsbedarf abbilden zu können, wurden Informationen zum subjektiven Gesundheitszustand (Wie würden Sie Ihren Gesundheitszustand im Allgemeinen beschreiben? Skala: Ausgezeichnet/sehr gut/gut/schlecht/sehr schlecht), zur Pflegebedürftigkeit (Pflegegrad 1-5), zum Unterstützungsbedarf und zur Versorgungssituation erhoben. Informationen zum kognitiven Status (MMSE, Mini Mental State Examination) wurden dem Datensatz der Hauptstudie „intersec-CM“ entnommen (Erhebungszeitpunkt 2018/2019). Des Weiteren wurde erfragt, ob im eigenen Umfeld jemand an Corona erkrankt ist.

***Postskript:*** Im Anschluss an jedes Interview wurden ein Postskript erstellt, in dem sämtliche Eindrücke, wie z.B. Gesprächsinhalte, Kontexteinflüsse, eigene Gedanken und spontane Interpretationsideen notiert wurden.

Die Daten wurden mittels inhaltlich strukturierender Inhaltsanalyse ausgewertet [5]. Gebildet wurden nach mehrfacher Materialdurchsicht folgende thematische Kategorien, die sich deduktiv aus dem Leitfaden und induktiv aus dem Material ergeben haben.

1. Corona-Pandemie allgemein
   1. Bewertungen/Assoziationen/Vergleiche
   2. Pandemiebezogene Sorgen/Gedanken allgemein
2. Bedrohungserleben/Ängste und Sorgen
   1. Objektive Gefährdung/Bedrohungspotenzial (Wissen)
   2. Einschätzung der subjektiven Gefährdung (eigene Corona-Erkrankung)
   3. Sorgen um Andere (Corona-Erkrankung)
   4. Folgen der Krise
   5. Erklärungsansätze/Hintergründe
   6. Eigene Sorgen/Herausforderungen (im Alltag)
3. Lebensalltag
   1. Allgemeine Einschätzung/Bewertung
   2. Alltagsversorgung und Haushaltsführung
   3. Gesundheitsversorgung und Pflege
   4. Sozialleben/Familie: Kontakte, Aktivitäten
   5. Zu Hause sein
   6. Kompensationsstrategien
4. Schutzmaßnahmen
   1. Einstellung zu geltenden Schutzmaßnahmen
   2. Persönliches Schutzverhalten
   3. Erfahrungen/Einschätzungen Verhalten anderer
   4. Beurteilung politischer Maßnahmen/Schutz für ältere Menschen
5. Information und Kommunikation
   1. Bewertung der erhaltenen Informationen (Politik und Medien)
   2. Informationsquellen
6. Hoffnungen/Wünsche

In einer tabellarischen Themenmatrix wurden die entsprechend codierten und paraphrasierten Textstellen (teilweise auch aussagekräftige Originalzitate) unter Beibehaltung des jeweiligen Fallbezugs geordnet und inhaltlich weiter konkretisiert und systematisiert. Die Auswertung erfolgte themenbasiert entlang der Kategorien und fallübergreifend, wobei die Antworten und Einschätzungen aller Studienteilnehmer*innen berücksichtigt wurden. Die Ergebnisdarstellung erfolgt anhand von sieben zentralen Themenbereichen.
