# Supplementary material for: “You have to take it that way.” A study of the subjective experience of the corona pandemic by older people in need of help and care living at home
Source: Z Gerontol Geriatr. 2021 Apr 15;54(4):359–64. [Article in German] doi: 10.1007/s00391-021-01888-6 (PMC8047544; doi:10.1007/s00391-021-01888-6)
Supplement: Supplementary file 2 [file 391_2021_1888_MOESM2_ESM.docx]

**„Das muss man so nehmen.“ Eine Studie zum subjektiven Erleben der Coronapandemie älterer hilfe- und pflegebedürftiger Menschen in der Häuslichkeit**

**"You have to take it that way." A study of the subjective experience of corona pandemic of older people in need of help and care living at home**

**Supplement: Stichprobenbeschreibung**

| **Tabelle: Stichprobenbeschreibung** | |
| --- | --- |
| **Befragte insgesamt** | 12 (n) |
| **Geschlecht** |  |
| Frauen | 9 |
| Männer | 3 |
| **Alter** |  |
| Altersspanne (Jahre) | 77 - 91 |
| Durchschnittsalter (Jahre) | 82,3 |
| Median | 80,0 |
| **Geburtsort** |  |
| Deutschland (vor und nach 1945) | 11 |
| Ausland | 1 |
| **Familienstand** |  |
| verheiratet | 4 |
| geschieden | 2 |
| verwitwet | 6 |
| **Kinder** |  |
| ja | 11 |
| nein | 1 |
| **Wohnsituation** |  |
| alleinlebend | 7 |
| mit Partner/in | 4 |
| Wohngemeinschaft | 1 |
| **Wohnort** |  |
| Alte Bundesländer | 9 |
| Neue Bundesländer | 3 |
| ländlicher Raum* | 3 |
| städtischer Raum* | 9 |
| **Schulbildung** |  |
| 7 bis 8 Jahre (Volksschule, Volksschulabschluss) | 7 |
| 10 Jahre (Mittlere Reife) | 3 |
| 12 Jahre** | 1 |
| keine Angabe | 1 |
| **Berufsausbildung** |  |
| ja | 8 |
| nein | 3 |
| keine Angabe | 1 |
| **Subjektiver Gesundheitszustand** |  |
| ausgezeichnet | 0 |
| sehr gut | 0 |
| gut | 2 |
| schlecht | 9 |
| sehr schlecht | 1 |
| **Kognitiver Status (MMSE)***** |  |
| Spannweite | 23-26 |
| Durchschnitt | 24,8 |
| Median | 25 |
| **Hilfe- und Pflegebedürftigkeit** |  |
| Hilfebedürftig**** | 4 |
| Pflegegrad 1 | 2 |
| Pflegegrad 2 | 4 |
| Pflegegrad 3 | 1 |
| Pflegegrad 4 | 1 |
| Pflegegrad 5 | 0 |
| **Versorgungsform (PG 2-5)** (n=6) |  |
| Pflegegeld | 3 |
| Pflegesachleistung (Ambulante Pflege) | 1 |
| Tages- und Nachtpflege | 2 |
| **Kennen Sie jemanden, der an Corona erkrankt ist?** |  |
| ja | 0 |
| nein | 12 |
| * Definition gemäß Raumabgrenzung des Bundesinstituts für Bau-, Stadt- und Raumforschung (BBSR)  ** im Ausland erworbener und nicht näher bezeichneter Schulabschluss  *** Erhebungszeitpunkt 2018/2019 während eines stationären Krankenhausaufenthaltes, Einschluss in die Hauptstudie „intersec-CM“ bei MMSE ≤ 26  **** Personen sind auf Hilfe und Unterstützung im Alltag angewiesen (z.B. Haushaltsführung, Mobilität), aber nicht formalrechtlich pflegebedürftig | |
